# Comparing public interest on stone disease between developed and underdeveloped nations: are search patterns on google trends similar?

**DOI:** 10.1590/S1677-5538.IBJU.2020.1076

**Published:** 2021-05-20

**Authors:** Giovanni S. Marchini, Kauy V. M. Faria, Felippe L. Neto, Fábio César Miranda Torricelli, Alexandre Danilovic, Fábio Carvalho Vicentini, Carlos A. Batagello, Miguel Srougi, Willaim C. Nahas, Eduardo Mazzucchi

**Affiliations:** 1 Faculdade de Medicina da Universidade de São Paulo Hospital das Clínicas Divisão de Urologia São PauloSP Brasil Seção de Endourologia, Divisão de Urologia, Hospital das Clínicas, Faculdade de Medicina da Universidade de São Paulo, SP, Brasil; 2 Glickman Urological and Kidney Institute Cleveland Clinic ClevelandOhio USA Glickman Urological and Kidney Institute, Cleveland Clinic, Cleveland, Ohio

**Keywords:** Lithiasis, Public Opinion, Epidemiology

## Abstract

**Objective:**

The big data provided by Google Trends may reveal patterns in health information-seeking behavior on population from Brazil and United States (US). Our objective was to explore and compare patterns of stone disease online information-seeking behaviors in both nations.

**Materials and Methods:**

To compare Relative Search Volume (RSV) among different urologic key words we chose “US” and “Brazil” as country and “01/01/2009 - 31/12/2018” as time-range. The final selection included 12 key words in each language. We defined “ureteroscopy” as a reference and compared RSV against it for each term. RSV was adjusted by the reference and normalized in a scale from 0-100. Trend presence was evaluated by Mann Kendall Test and magnitude by Sen's Slope (SS) Estimator.

**Results:**

We found an upward trend (p <0.01) in most of the researched terms in both countries. Higher temporal trends were seen for “Kidney Stone” (SS=0.36), “Kidney Pain” (SS=0.39) and “Tamsulosin” (SS=0.21) in the US. Technical treatment terms had little search volumes and no increasing trend. “Kidney Stent” and “Double J” had a significant increase in search trend over time and had a relevant search volume overall in 2018. In Brazil, “Calculo Renal”, “Colica Renal”, “Dor no Rim” and “Pedra no Rim” had a significant increase in RSV (p <0.001). More common and popular terms as “Kidney Stent” and “Tamsulosin” were highly correlated with “Kidney Pain” and “Kidney Stone” in both countries.

**Conclusions:**

In the last decade, there was a significant increase in online search for medical information related to stone-disease. Population from both countries tend to look more for generic terms related to symptoms, the disease, medical management and kidney stent, than for technical treatment vocabulary.

## INTRODUCTION

The prevalence of urinary stone disease has significantly increased worldwide in the last decades, with an overall prevalence of 7% to 12% (1–4). In Brazil, the number of stone-related hospitalizations increased from 58.165 in 1998 to 67.306 in 2012 (5, 6). As the disease becomes more prevalent, there is an increase in patient's interest which is translated in a rise on the volume of search for information regarding the matter globally (7).

According to the Statista Research Department (8), which include nearly 210 million individuals and 140 million internet users in 2016, Brazil is the largest internet market in Latin America and the fourth largest internet market in the World when considering the number of internet users (9). The United States, the fourth largest country in the World by land area, is no exception. With over 312 million internet users as of 2018, it is one of the largest online markets worldwide. Internet usage in the United States is frequent, with 43 percent of surveyed adults saying that they use the internet several times a day as of February 2018, compared to just eight percent who said they accessed the internet about once a day (10).

In the last decade, a new discipline has emerged in order to study the determinants and distribution of health information on the internet, named infodemiology. It aims to monitor health seeking behavioral patterns, epidemiology, etiology, and treatment of various medical conditions worldwide by using online monitoring tools. Google Trends (GT) is one of the most robust of these platforms, in which internet quests are catalogued and the combined information made public (11, 12). Few studies have investigated online trends regarding stone disease (7, 11).

The big data provided by GT can reveal patterns in health information-seeking behavior on population from Brazil and US, allowing development of target information to the public and comparison between countries. The aim of this study was to analyze patterns of stone disease information-seeking behaviors in Brazil and the US.

## MATERIALS AND METHODS

### 

#### Data Acquisition and Interpretation

GT is a web-service offered by Google Inc. that keeps track of online key words interest accordingly to country or region over a selected time period (12, 13). In addition, the search of different terms in different regions can be compared simultaneously. Data is downloaded from the Web in “csv” format and adjusted as follows: search results are proportionate to the time and location of a query, each data point is divided by the total absolute searches of the geography and time range it represents, to compare relative popularity. Otherwise places with the most search volume would always be ranked highest. The resulting numbers are then scaled on a range of 0 to 100 based on a topic's proportion to all searches on all topics. Different regions that show the same number of searches for a term will not always have the same total absolute search volumes.

GT allows for historical trend analysis of the seeking pattern (12) and provides a Relative Search Volume (RSV) which is a sampled estimate of a particular query share according to location and time normalized by the highest query of the period in a 1-100 scale. Multiple terms analysis is allowed for query comparison.

#### Data Collection and Analysis

We have downloaded data from GT on August 11^th^, 22^th^ and 24^th^ 2019. To compare RSV among different urologic key words we have used “United States” and “Brazil” as country, “01/01/2009 - 31/12/2018” as time-range, “All Categories” as category and “Web Search” as type of search.

English terms were first selected after a recent study (11) which explored their popularity and the appropriate counterpart's words in Portuguese were chosen by an endourology expert (GSM) to make sure both languages would have similar meanings and translations. We aimed to include medical terms and non-medical terms in other to evaluate which ones were of most importance in the platform. When two similar terms were compared, we only considered the most relevant one in the platform in the analysis to avoid redundances. The final key words for each treatment were chosen based on multiple attempts until one was found to capture the greatest RSV for the period. The final comprehensive selection included 24 key words, 12 in each language:

- US: Kidney stone; Renal stone; Kidney stone surgery; Renal colic; Kidney pain; Ureteroscopy (URS); Extracorporeal shockwave lithotripsy (ESWL); Percutaneous nephrolithotomy (PCNL); Tamsulosin; Kidney stent (more relevant than double J in terms of RSV); Lithotripsy; Laser lithotripsy.
- Brazil: Pedra de rim; Cálculo renal; Cirurgia pedra no rim; Colica renal; Dor no rim; Ureteroscopia; Litotripsia extracorporea; Nefrolitotripsia percutanea; Tansulosina; Duplo J (more relevant than “Cateter renal”); Litotripsia; Litotripsia a laser.

### Statistical Analysis

To compare more than GT's limit of five treatments, we defined “ureteroscopy” (in both languages: “ureteroscopy” for the US; “ureteroscopia” for Brazil) as a reference and downloaded RSV comparisons against it for each term. We adjusted the RSV numbers by the reference and normalized them by the highest RSV for the period in a scale from 0-100.

Trend presence was evaluated using the Mann Kendall Test and magnitude was estimated using the Sen's Slope (SS) Estimator. Both of them apply to non-parametric data. Correlation analysis was done using Pearson method, which is the standard used by Google in Correlate Service. All statistical analysis was done in R version 3.5.1. Significance was set at p <0.05.

## RESULTS

### 

#### Temporal Trend Analysis

**Figure 1:**
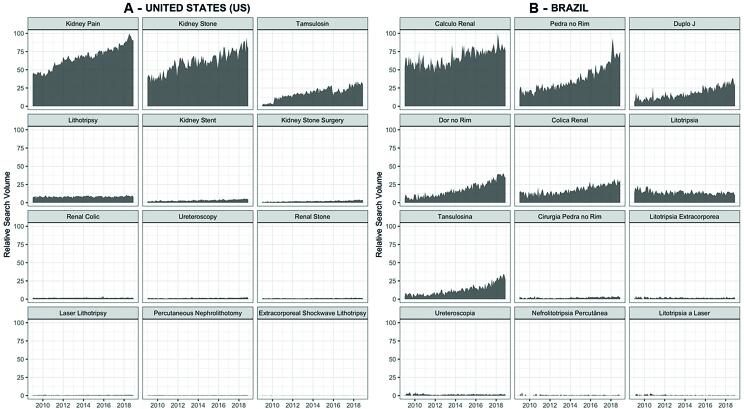
Dark area of the graphic express the Relative Search Volume (RSV) in United States - US (A) and Brazil (B) between 2009 and 2018 for 12 terms related to stone disease.

There was an increase in the volume of researched terms in both countries (p-value <0.01; table-1). The RSV over time for US and Brazil for each search term is depicted in Figure-1.

**Table 1:** Total Relative Search Volume (RSV) in United States (US) and Brazil in 2009 and 2018, trend variation measured by Mann Kendall Sen's Slope (SS) Estimator, and term correlation between (R) countries.

Brazil Queries						United States Queries				Brazil x US	
Search Term	Sens Slope	Mann Kendall	Mean RSV		Search Term	Sens Slope	Mann Kendall	Mean RSV		Correlation (R)	p-value
		p-value	2009	2018			p-value	2009	2018		
“Pedra No Rim”	**0.408**	**<0.001**	20.15	72.89	Kidney Stone	**0.369**	**<0.001**	38.77	82.93	0.81	**<0.001**
“Dor No Rim”	**0.265**	**<0.001**	6.63	37.07	Kidney Pain	**0.398**	**<0.001**	44.66	92.08	0.93	**<0.001**
“Calculo Renal”	**0.247**	**<0.001**	59.75	81.91	Renal Stone	**0.005**	**<0.001**	1.03	1.60	0.45	**<0.001**
“Tansulosina”	**0.178**	**<0.001**	6.23	26.63	Tamsulosin	**0.215**	**<0.001**	3.07	30.70	0.81	**<0.001**
“Colica Renal”	**0.160**	**<0.001**	12.67	28.25	Renal Colic	0.001	0.10	1.62	1.88	0.19	**<0.05**
“Cirurgia Renal”	**0.015**	**<0.001**	1.67	3.12	Kidney Stone Surgery	**0.019**	**<0.001**	1.16	3.61	0.50	**<0.001**
“Nefrolitotripsia percutânea”	0.001	0.37	0.74	0.59	Percutaneous nephrolithotomy	0.000	0.27	0.45	0.53	0.05	0.57
“Ureteroscopia”	-0.001	0.62	2.02	1.60	Ureteroscopy	**0.008**	**<0.001**	1.29	2.14	-0.03	0.73
“Litotripsia extracorpórea”	-0.003	0.11	1.9	1.90	Extracorporeal shockwave lithotripsy	0.000	0.30	0.16	0.08	-0.32	**<0.001**
“Laser”	**-0.003**	**<0.01**	0.71	0.44	Laser lithotripsy	**0.002**	**<0.001**	0.58	0.08	-0.08	0.38
“Litotripsia a laser”	**-0.032**	**<0.001**	17.5	13.25	Lithotripsy	**0.000**	**<0.01**	8.0	9.08	0.15	0.15
“Duplo J”	**0.200**	**<0.001**	9.56	32.31	Kidney Stent	**0.025**	**<0.001**	1.96	4.97	0.78	**<0.001**

“Pedra de rim” =; “Calculo renal” = Renal stone; “Cirurgia pedra no rim” = Kidney stone surgery; “Colica renal” = Renal colic; “Dor no rim” = Kidney pain; “Ureteroscopia” = Ureteroscopy; “Litotripsia extracorpórea” = Extracorporeal shockwave lithotripsy; “Nefrolitotripsia percutânea” = Percutaneous nephrolithotomy; “Tamsulosina” = Tamsulosin; “Duplo J” = Kidney stent; “Litotripsia” = Lithotripsy; “Litotripsia a laser” = Laser lithotripsy.

In 2018, terms related to general symptoms or more generic expressions, e.g. “Kidney Stone” and “Kidney Pain”, had higher trends as measured by Sens's Slope and were the most searched group by US internet users. Specifically, the highest temporal trends were seen for “Kidney Stone” (SS=0.36), “Kidney Pain” (SS=0.39) and “Tamsulosin” (SS=0.21) in the US (Table-1). “Tamsulosin” had an expressive increase in search volume and achieved in 2018 an expressive RSV of 30.70. Interestingly, “Renal Colic” and “Renal Stone” had low search volumes.

Expressions related to stone disease therapies as “ESWL”, “Laser lithotripsy”, “PCNL” and its Portuguese counterparts had little search volumes and no increasing trend, remaining low in public interest during the ten-year analysis (Table-1). In regard to the surgical treatment terms, the most looked up was “Kidney Stent” which was close to 10% of the related search on pharmacological treatments represented by “Tamsulosin”. “Kidney Stent” had a significant increase in search trend over time and had a relevant search volume overall in 2018 (Figure-1, Table-1). “Kidney Stone Surgery”, a more general expression, showed a significant increase over time (SS=0.019, p <0.001).

In Brazil, generic and clinical terms as “Calculo Renal” (renal stone), “Colica Renal” (renal colic), “Dor no Rim” (kidney pain) and “Pedra no Rim” (kidney stone) had noteworthy search volumes in the studied period and a significant increase in RSV (p <0.001). However, different from US, “Duplo J”, the counterpart for “Kidney Stent”, had higher trends (SS=0.20, p <0.001) compared to the pharmacological treatment represented by “Tamsulosina” (SS=0.17, p <0.001). Even so, in resemblance to the US, “Tamsulosina” also had an expressive search volume in 2018 (RSV=26.63) and showed a significant 4.2-fold increase from 2009 (RSV=6.23). Specific surgical terms had low search volumes and no increase in trend in similarity to the US (Figure-1, Table-1). In addition, the terms “Laser” (SS=-0.003, p <0.01) and “Litotripsia a laser” (laser lithotripsy, SS=-0.032, p <0.001) showed a significant decrease in mean search volumes over time.

#### Correlation Analysis

When comparing corresponding terms between US and Brazil, strong positive correlations (Pearson correlation >0.7) were found for the following pairs: “Kidney Stone” and “Pedra no Rim” (R=0.81, p <0.001); “Kidney Pain” and “Dor no Rim” (R=0.93, p <0.001); “Tamsulosin” and “Tamsulosina” (R=0.81; p <0.001); “Kidney Stent” and “Duplo J” (R=0.78; p <0.001) (Table-1).

**Figure 2:**
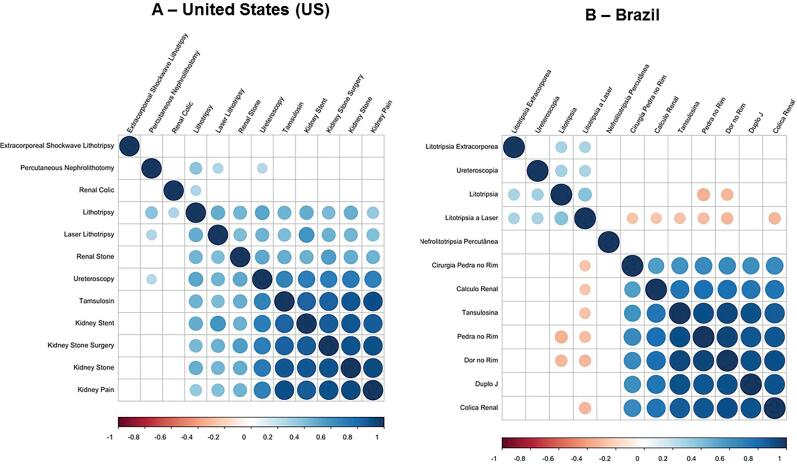
Correlation evaluation between stone-disease related terms searched on Google Trends platform in the United States - US (A) and Brazil (B).

Figures 2A and 2B depict independent term's correlations in US and Brazil, respectively. For positive correlations, a blue circle matching the terms in left column and upper row was used. The more intense and the larger the circle, the higher the correlation. A red circle was used when no correlation was found between terms.

The correlation is positive when there is a blue circle matching the terms in left column and upper row. When the color is more intense and the circle larger, the correlationa is higher. The red circle is interpreted as no correlation between terms.

Correlation for each country separately showed similar patterns in which terms with more medical knowledge content as “ESWL”, “PCNL”, “Lithotripsy” had lower correlation with popular terms as “Kidney Pain” or “Kidney Stone”. A more generic surgical term, “Kidney Stone Surgery”, had higher correlation as compared to the treatments above mentioned. More common and popular terms as “Kidney Stent” and “Tamsulosin” were highly correlated with “Kidney Pain” and “Kidney Stone” in both countries.

## DISCUSSION

We have previously evaluated GT patterns for stone disease in the US (5) but not in Brazil. In the present study, we could notice a remarkable increase in interest of the patients in regard to medical information related to renal stones in both countries. Even though Brazil and US are in distinguish stages of economic development, the growths were correlated and occurred in a similar pattern. This may indicate a World trend in the use of internet engines as the main source for health information seeking for urinary stone disease. Nonetheless, our data suggest a somewhat different research pattern in each country. This may be related to cultural and linguistic differences between nations.

The GT platform does not allow for understanding which term was looked for first by an individual. Nevertheless, the volume of search allows for some hypothesis. In Brazil, patients probably start their research by symptoms and causes of the disease, e.g. “Dor no rim” (“Kidney pain”), “Cólica renal” (“Renal colic”). After that, they possibly look for information regarding potential clinical and surgical treatments. As shown in Figure-2, patients in Brazil seeking for “Dor no rim” (“Kidney pain”), “Cólica renal” (“Renal colic”) and “Pedra no rim” (“Kidney stone”) are also commonly looking for “Double J” (“Kidney stent”), “Tamsulosina” (“Tamsulosin”) and “Cirurgia pedra no rim” (“Kidney stone surgery”). Specific surgical terms are not frequently searched for. This means they are seeking for solutions for their problems in their own vocabulary. Remarkably, tamsulosin is still considered an off-label drug for stone disease and it is mainly used for benign prostatic hyperplasia and lower urinary trac symptoms. Still, we did not want to leave the term out of our search since it is routinely used for medical expulsive therapy and for relief of stent-related symptoms. The finding that “Kidney Stent” and “Tamsulosin” were highly correlated with “Kidney Pain” and “Kidney Stone” in both countries suggests that those terms were being searched for at the same time by several individuals in the same situation. Nonetheless, data must be evaluated with caution.

In the US, we found a similar web exploration pattern to Brazil. A specific difference was related to the terms “Renal colic” and “Renal stone”, much less used than “Kidney stone” and “Kidney pain” and also their counterparts in Brazil. Nonetheless, overall, the population mainly search for symptoms and the disease itself and does not focus on specific urologic treatment terms. Noteworthy, technical surgical terms are the ones usually used by urologists and experts when making public statements, giving interviews and/or writing posts for invited or their own websites. This must be taken into consideration when preparing health related campaigns regarding stone disease. Otherwise, the information will just not be found by the public. Our previous study has shown that there is a discrepancy between medical publications on Pubmed and GT searched terms (7). This brings up two important questions: first, are we studying and publishing in what is really affecting patient's lives? Mainly diet, lifestyle and preventing kidney stones and renal colic burden. Or are we more focused on improving in the way we treat the consequences? Secondly, even if less invasive surgical modalities are the core for patients, are we communicating in an effective manner? Whilst we do not have the answers, we should seek and embrace a patient-centered approach.

Dreher et al. were the first authors to look at GT platform in regard to kidney stones (11). By using terminology related to kidney stone surgical intervention in English within the US in a 6-year period (2011-2017), they found “Kidney stone surgery” as the most common term in comparison to “PCNL”, “ESWL”, “URS” and “Laser Lithotripsy”. In discrepancy to our study, the authors concluded that research trends for the term “Kidney stone surgery” remained stable over time. Three key aspects might explain why their results are in large different from ours. First, a shorter period of time was considered in their study. Second, they did not use statistical analysis to ensure their visual graphic impression. Finally, the authors did not look at expressions we found to be the more important addressed by the population, namely the ones related to the disease itself.

Wu et al evaluated the global public interest in rheumatoid arthritis by evaluating search term popularity changes of the disease on GT over a decade and found a significant seasonal variation, with a peak in April (14). In addition, the authors underline that physicians could use the top rising search queries to better indicate specific online sources of reliable information for patients. Our analysis showed an increased interest in stone-related terms. However, a prominent seasonal variation was not clearly found. Although it is common sense that the incidence of renal colic is higher in high temperature regions and/or months, both analyzed countries are continental, and this impact could have been diluted in the overall evaluation. Nevertheless, in our previous analysis (7), we could demonstrate that US states with hotter weather had a significant higher interest on the term “kidney stone” than colder states.

With the data gathered in this study we may better elaborate content to patients seeking for information regarding stone disease in Brazil. The key words “Calculo Renal” (renal stone), “Colica Renal” (renal colic), “Dor no Rim” (kidney pain), “Pedra no Rim” (kidney stone) and “Duplo J” (Kidney Stent) should be the ones chosen when planning public strategies to educate the population nationwide. Prevention campaigns might focus on those key words and its effectiveness could be monitored continuously in the same online platform. Urologists and epidemiologists should not use medical surgical terms on online campaigns. Furthermore, as seen for other diseases, online search volumes could be gathered with other information related to stone disease, e.g. air humidity and mean temperature, to enhance forecast epidemiology of this prevalent disease. Finally, by studying what patients really seek for, urological medical community could aim efforts to improve those areas rather than to what the surgeon believe is more important for the patient.

Our study has several limitations. First, although it reflects the online search patterns of countries with expressive internet usage, there are significant restrictions for use of the internet in poor areas of both nations. Nonetheless, both are democratic nations with no political or dictatorial restrictions in that matter. Secondly, the database does not allow for granular information. And even though we used a trend statistical analysis tool, the yearly number of search volume reflects a transversal study in each analysed data point. Third, the order in which individuals seek for terms are not completely known. By analysing related terms, we might better understand the populational behaviour but there is no formal manner to infer which term was looked for first. Fourth, the potential benefits of using the data acquired in this study are still to be tested in public strategies for stone disease prevention and educational purposes. Fifth, the analysis of terms not specific to stone disease, e.g. tamsulosin, should be done cautiously as previously discussed. Last, data from Brazil and the US cannot be extrapolated to other countries. The same research should be performed in other geographic locations in order to attain specific results.

## CONCLUSION

In the last decade, there was a significant increase in online search for medical information related to stone-disease. Population from US and Brazil tend to look for terms related to symptoms and the disease itself. Also, medical management and kidney stent are expressions of special interest in both countries. On the contrary, technical terms of urologic procedures do not arouse interest to patients. Our findings are obvious to some extent but highlight the importance of choosing wisely which terms to use when elaboration public educational health campaigns related to stone disease. Prediction models for stone disease outbreaks are a line of investigation and could be added to the climate influence patterns already established.

## ABBREVIATIONS

GT: Google Trends
PCNL: Percutaneous Nephrolithotomy
RSV: Relative Search Volume
SS: Sen's Slope
SWL: Shockwave Lithotripsy
URS: Ureteroscopy
US: United States

## References

[1] 1. Sakhaee K. Pharmacology of stone disease. Adv Chronic Kidney Dis. 2009; 16:30-8. 10.1053/j.ackd.2008.10.004 PMC3088503 19095203

[2] 2. Moe OW. Kidney stones: pathophysiology and medical management. Lancet. 2006; 367:333-44. 10.1016/S0140-6736(06)68071-9 16443041

[3] 3. Scales CD Jr, Smith AC, Hanley JM, Saigal CS; Urologic Diseases in America Project. Prevalence of kidney stones in the United States. Eur Urol. 2012; 62:160-5. 10.1016/j.eururo.2012.03.052 PMC3362665 22498635

[4] 4. Wang S, Zhang Y, Zhang X, Tang Y, Li J. Upper urinary tract stone compositions: the role of age and gender. Int Braz J Urol. 2020; 46:70-80. 10.1590/S1677-5538.IBJU.2019.0278 PMC6968895 31851461

[5] 5. Danilovic A, Ferreira TAC, Maia GVA, Torricelli FCM, Mazzucchi E, Nahas WC, et al. Predictors of surgical complications of nephrectomy for urolithiasis. Int Braz J Urol. 2019; 45:100-7. 10.1590/S1677-5538.IBJU.2018.0246 PMC6442129 30521174

[6] 6. Marchini GS, Mello MF, Levy R, Vicentini FC, Torricelli FC, Eluf-Neto J, et al. Contemporary Trends of Inpatient Surgical Management of Stone Disease: National Analysis in an Economic Growth Scenario. J Endourol. 2015; 29:956-62. 10.1089/end.2015.0021 25706608

[7] 7. Marchini GS, Faria KVM, Neto FL, Torricelli FCM, Danilovic A, Vicentini FC, et al. Understanding urologic scientific publication patterns and general public interests on stone disease: lessons learned from big data platforms. World J Urol. 2020:1–7. 10.1007/s00345-020-03477-5 PMC7590553 33108478

[8] 8. [No Authors]. Statista Research Department. Global No.1 Business Data Platform. Statista. [Internet]. Available at. < https://www.statista.com/>

[9] 9. Navarro J. G. Internet usage in Brazil – statistics & facts Internet usage in Brazil. Statista. [Internet]. Available at. <https://www.statista.com/topics/2045/internet-usage-in-brazil/>. Accessed in 7, 2020.

[10] 10. Johnson J. Internet usage in the United States - Statistics & Facts. Statista. [Internet]. Available at. <https://www.statista.com/topics/2237/internet-usage-in-the-united-states/> Accessed in 4, 2021.

[11] 11. Dreher PC, Tong C, Ghiraldi E, Friedlander JI. Use of Google Trends to Track Online Behavior and Interest in Kidney Stone Surgery. Urology. 2018; 121:74-8. 10.1016/j.urology.2018.05.040 30076945

[12] 12. Mavragani A, Ochoa G, Tsagarakis KP. Assessing the Methods, Tools, and Statistical Approaches in Google Trends Research: Systematic Review. J Med Internet Res. 2018; 20:e270. 10.2196/jmir.9366 PMC6246971 30401664

[13] 13. Rogers S. What is Google Trends data - and what does it mean? Google News Labs. [Internet]. Available at. < https://medium.com/google-news-lab/what-is-google-trends-data-and-what-does-it-mean-b48f07342ee8> Accessed in 6, 2016.

[14] 14. Wu GC, Tao SS, Zhao CN, Mao YM, Wu Q, Dan YL, et al. Leveraging Google Trends to investigate the global public interest in rheumatoid arthritis. Rheumatol Int. 2019; 39:1439-44. Erratum in: Rheumatol Int. 2019; 39:1445. 10.1007/s00296-019-04297-6 30955063

